# Evidence for association between the HLA-DQA locus and abdominal aortic aneurysms in the Belgian population: a case control study

**DOI:** 10.1186/1471-2350-7-67

**Published:** 2006-07-31

**Authors:** Toru Ogata, Lucie Gregoire, Katrina AB Goddard, Magdalena Skunca, Gerard Tromp, Wayne D Lancaster, Antonio R Parrado, Qing Lu, Hidenori Shibamura, Natzi Sakalihasan, Raymond Limet, Gerald L MacKean, Claudette Arthur, Taijiro Sueda, Helena Kuivaniemi

**Affiliations:** 1 Center for Molecular Medicine and Genetics, Wayne State University School of Medicine, Detroit, Michigan, USA; 2 Department of Immunology and Microbiology, Wayne State University School of Medicine, Detroit, Michigan, USA; 3 Department of Epidemiology and Biostatistics, Case Western Reserve University, Cleveland, Ohio, USA; 4 Department of Obstetrics and Gynecology, Wayne State University School of Medicine, Detroit, Michigan, USA; 5 Department of Cardiovascular Surgery, University of Liège, Liège, Belgium; 6 Department of Surgery, Dalhousie University, Halifax, Canada; 7 Department of Surgery, Graduate School of Biomedical Science, Hiroshima University, Hiroshima, Japan; 8 Department of Surgery, Wayne State University School of Medicine, Detroit, Michigan, USA

## Abstract

**Background:**

Chronic inflammation and autoimmunity likely contribute to the pathogenesis of abdominal aortic aneurysms (AAAs). The aim of this study was to investigate the role of autoimmunity in the etiology of AAAs using a genetic association study approach with HLA polymorphisms.

**Methods:**

HLA-DQA1, -DQB1, -DRB1 and -DRB3-5 alleles were determined in 387 AAA cases (180 Belgian and 207 Canadian) and 426 controls (269 Belgian and 157 Canadian) by a PCR and single-strand oligonucleotide probe hybridization assay.

**Results:**

We observed a potential association with the HLA-DQA1 locus among Belgian males (empirical p = 0.027, asymptotic p = 0.071). Specifically, there was a significant difference in the HLA-DQA1*0102 allele frequencies between AAA cases (67/322 alleles, 20.8%) and controls (44/356 alleles, 12.4%) in Belgian males (empirical p = 0.019, asymptotic p = 0.003). In haplotype analyses, marginally significant association was found between AAA and haplotype HLA-DQA1-DRB1 (p = 0.049 with global score statistics and p = 0.002 with haplotype-specific score statistics).

**Conclusion:**

This study showed potential evidence that the HLA-DQA1 locus harbors a genetic risk factor for AAAs suggesting that autoimmunity plays a role in the pathogenesis of AAAs.

## Background

Several distinct processes contribute to the pathologic changes observed in abdominal aortic aneurysms (AAAs). The most apparent of these are chronic inflammation, destructive remodeling of the extracellular matrix, and depletion of vascular smooth muscle cells [1]. Local immune responses in the aorta are an important factor in AAA pathogenesis. Autoimmunity has been proposed to play a role in the pathogenesis of AAA [2,3]. Infiltration of monocytes, macrophages, B-lymphocytes, plasma cells and T-lymphocytes (including both CD4 and CD8 T-cells) is commonly observed in the AAA walls [4]. Although the actual factors (triggers) responsible for initiating the chronic inflammatory response in the pathogenesis of AAA are not yet known, HLA loci, particularly the HLA-DQ and HLA-DR antigens, may play a key role.

The major histocompatibility complex (MHC) is located at chromosome 6p21.31 and is the most gene-dense and polymorphic region of the human genome identified so far [5]. Historically, the MHC has been divided into three regions: HLA class I, class II and class III. Although class I antigens are present on the surface of most types of cells in the human body, class II antigens are expressed by a few types of antigen-presenting cells, namely B-lymphocytes, macrophages, dendritic cells, thymic epithelial cells, and activated T lymphocytes [6,7]. The MHC locus has been associated with more diseases than any other region of the human genome with more than 20,000 research articles published [8], and most of the significant associations were with the class II polymorphisms [5,7].

The HLA class II region contains five isotypes, HLA-DM, -DO, -DP, -DQ and -DR, all of which are heterodimers composed of α and β chains [7]. There are only few polymorphisms in the HLA-DM and -DO isoforms. On the other hand, HLA-DP, -DQ and -DR are quite polymorphic [7]. For HLA-DQ, both the α and β chains, which are expressed by HLA-DQA1 and -DQB1 genes, respectively, contribute to the variability. For HLA-DRB, only the β chain, which is expressed by the HLA-DRB1 gene, contributes to the variability [7]. HLA-DRB1 (β chain) has another functional isoform, DRB3-5, whose genes are located close to the -DRB1 gene. Only one allele in each individual is expressed from DRB3, 4 and 5 genes combined [7]. HLA-DQ and -DR proteins are responsible for presenting foreign peptide antigens from infectious agents, such as bacteria, viruses or autoimmune antigens, to CD4 T-cells. These antigens stimulate CD4 T-cell responses that activate B-cells and macrophages. The structure of the HLA-DQ or -DR peptide-binding groove varies considerably depending on which DQA1, DQB1 and DRB1 alleles are being exposed [9]. These genetic differences may affect the immune response by increasing or decreasing the ability of HLA-DQ or -DR molecules to bind and properly present foreign antigens to the CD4 T-cell [7,10].

Several association studies between HLA polymorphisms and AAAs have been performed [11-19]. The sample sizes of these studies, however, were small and the results were inconsistent. The aim of the current study was to investigate further the role of autoimmunity in the etiology of AAAs by carrying out a genetic association study with the HLA-DQA1, -DQB1, and -DRB polymorphisms for AAA.

## Methods

### Study population

A definition of AAA by Johnston et al [20] (a diameter of infrarenal aorta ≥ 3 cm) was used. These standards have also been used by other investigators [21-23]. Altogether 387 unrelated AAA cases (males: 316, 81.7%), 180 Belgian (males: 161, 89.4%), admitted to University Hospital of Liège in Liège, and 207 Canadian (males: 155, 74.9%), admitted to Dalhousie University Hospital in Halifax, were entered into the study. Seventeen patients were admitted for emergency repair of ruptured AAA and 335 patients were admitted for elective surgery. Thirty-five patients were diagnosed with AAA using ultrasonography and were not operated on due to old age or because the size of the aneurysm was relatively small (< 5 cm). Altogether 152 cases (39.3%) had a family history of AAA, which was defined as having at least one first-degree relative affected with AAA. All patients were Caucasian.

Control samples were obtained from 426 Caucasians (males: n = 217, 50.9%; 269 Belgian and 157 Canadian) and included spouses of AAA cases (n = 114; all Canadian; 4 males, 110 females), or individuals admitted to the same hospitals for reasons other than AAA (n = 312; 183 Belgian males, 86 Belgian females; 29 Canadian males and 14 Canadian females).

The study was approved by the Institutional Review Boards of Wayne State University School of Medicine and each patient recruiting center. All subjects gave informed written consent to participate into the study.

### Genotyping

Genomic DNA was isolated from peripheral blood using a Puregene kit (Gentra Systems, Minneapolis, MN). Before performing genotyping PCR, a whole-genome amplification using primer extension preamplification (PEP) was carried out to increase the amount of template DNA available for genotyping and to ensure that limited resources are used cost-effectively [24]. The performance of PEP in subsequent genotyping reactions was validated extensively in our laboratory previously [24]. The PEP products were diluted 100-fold and used for genotyping. Throughout the manuscript we use the gene symbols approved by the HUGO Gene Nomenclature Committee [25].

HLA-DQA1, -DQB1, -DRB1 and -DRB3-5 alleles were determined by PCR and single-strand oligonucleotide probe hybridization assay. HLA-DQA1 alleles were determined according to Kimura et al [26]. Quality control samples, including positive and negative controls, were included in each set of 96 samples run in genotyping reactions. Positive controls included samples whose HLA genotypes were known. HLA-DQA1 was amplified using primers DQAP1 (5'-ATGGTGTAAACTTGTACCAGT-3') and DQAP2 (5'-TTGGTAGCAGCGGTAGAGTTG-3') to generate a 230-bp fragment. Fourteen single-strand oligonucleotide probes discriminated between HLA-DQA1*0101/0104, *0102, *0103, *0201, *03011/0302, *03012, *0401, *0501 and *0601 alleles. HLA-DQB1 alleles were determined as previously described [27]. HLA-DQB1 was amplified using primers DQP14 (5'-TGTGCTACTTCACCAACGGG-3') and DQP5 (5'-GGTAGTTGTGTCTGCACAC-3') to generate a 210-bp fragment. Twenty-five single-strand oligonucleotide probes could discriminate between HLA-DQB1*0201/0202, *0301, *0302, *03031, *03032 *0304, *0305, *0401, *0402, *0501, *0502, *0503, *0504, *0601, *0602, *0603, *0604, *0605, *0606, *0607 and *0608 alleles. Both HLA-DRB1 and DRB3-5 alleles were amplified using primers GH46 (5'-CCGGATCCTTCGTGTCCCCACAGCACG-3') and P2A (5'-TCGCCGCTGCACTGTGAAGC-3') to generate a 290-bp fragment. Forty single-strand oligonucleotide probes could discriminate between HLA-DRB1*0101, *0102, *0103, *0301/0303, *0302, *0401, *0402, *0403, *0404, *0405, *0406, *0407, *0408, *0409, *0410, *0411, *0412, *0413, *0414, *0415, *0701/0702, *0801, *0802, *0803, *0804, *0805, *0806, *0901, *1001, *1101, *1102, *1103, *1104, *1105, *1201, *1202, *1301, *1302, *1303, *1304, *1305, *1306, *1401, *1402, *1403, *1404, *1405, *1406, *1407, *1408, *1409, *1410, *1501, *1502, *1503, *1601 and *1602 as well as DRB3*0101, *0201, *0202, *0301, DRB4*0101, DRB5*0101, *0102, *0201/0202 and *0203 alleles [26]. Samples whose hybridization pattern failed to match with the pattern of a known allele were classified as "could not be determined". Exceptions were the unknown alleles which could be classified into two possible alleles, such as HLA-DRB1*0403 or *0407, but could not be classified into one particular allele, and were then designated as "*0403 or *0407". Two individuals (T.O. and L.G.) scored the results independently and discrepancies were resolved by re-evaluation of the raw data or repeating the experiment, if necessary.

### Statistical analyses

First, the distributions of allele frequencies between the Belgian and Canadian control groups were compared. Asymptotic p-values were obtained using the χ^2 ^test for association. Because of the large number of alleles at each locus, we also obtained empirical p-values for the test of association for each allele using a permutation test for the entire table (called the T1 test statistic as opposed to other test statistics computed by this program that collapses the table in various ways) as implemented in the computer program CLUMP [28]. In this permutation test, the population group is permuted numerous times, and the test statistic is recomputed for each permutation. The observed test statistic is then compared to the distribution of the permuted test statistics to obtain an empirical p-value. Significant differences between the two control groups for all of the HLA loci were found. The p-values were: 0.062 (empirical: 0.056) for DQA; 0 (empirical: 0.0001) for DQB, 0.008 (empirical: 0.0046) for DRB, and 0 (empirical: 0.0001) for DRB3-5. Canadian and Belgian populations were, therefore, analyzed separately. To evaluate the association between alleles and AAA, the same approach was used. The Haplo.stats program [29] was used to estimate haplotype frequencies via the expectation-maximization (EM) algorithm for HLA-DQA1, -DQB1, -DRB1 and -DRB3-5 alleles, and to compute two score tests of association between disease status and HLA haplotype: 1) global score statistics and 2) haplotype-specific score statistics. Rare haplotypes with frequencies ≤ 0.01 were pooled into a single baseline group. The score test is developed under the generalized linear model framework, where the score function is the first derivative of the log-likelihood from the exponential family distribution. The variance matrix is determined by the matrix of negative second partial derivative of the log-likelihood. Using the results from the EM algorithm, the score statistics *S *= *U*^*T*^*V*^-1^*U *are evaluated under the null hypothesis, which asymptotically has a chi-square distribution. Empirical p-values are obtained for the score statistics using permutation. Results on haplotypes with only two loci are reported, since the contingency table for 3- or 4-locus results was sparse given the large number of alleles at each locus. Analyses were also conducted stratified by sex, and the presence of a positive family history. Pairwise measures of linkage disequilibrium (LD) were estimated using the observed genotypes for the four HLA loci using the GOLD program [30]. All measures of D' were between 0.26 and 0.37, indicating comparatively low LD between loci. Correction for multiple testing was applied to the most significant p-values using the Sidak function.

### Post hoc power calculations

Since the HLA locus has a large number of alleles and the allele frequencies are variable across populations, we computed power for the locus based on the frequency observed in the samples. Power calculations were performed using the power calculator for binomial proportions in Splus 7.0 (Insightful Corporation, Seattle, WA) for a test of the null hypothesis that p1 = p2, where p1 is the allele frequency in cases and p2 is the allele frequency in controls, versus the alternative hypothesis that the allele frequencies for the cases and controls are different. To obtain 80% power with the risk allele frequency of 0.208 in the cases and 0.124 in the controls, we needed 331 subjects per group for an α of 0.05, and 460 subjects per group for an α of 0.0125 (to account for multiple testing).

## Results

HLA-DQA1, -DQB1, -DRB1 and -DRB3-5 allele frequencies are summarized in Tables 1, 2, 3, 4. Empirical p-values obtained by permutation test and asymptotic p-values obtained by χ^2 ^test are shown in Table 5. Results are shown separately for the Belgian and Canadian populations for two reasons: 1) comparison of the distribution of allele frequencies between the Belgian and Canadian control groups using a chi-square test showed significant differences for all of the HLA loci. The p-values were: 0.062 (empirical: 0.056) for DQA; 0 (empirical: 0.0001) for DQB, 0.008 (empirical: 0.0046) for DRB, and 0 (empirical: 0.0001) for DRB3-5; and 2) in our previous work we showed highly significant departure from Hardy-Weinberg proportions for several markers in the combined sample, but not in the subsets, indicating evidence for the presence of population stratification [31]. There was a nominally significant association between AAA and HLA-DQA1 locus in the Belgian population (empirical p = 0.039; asymptotic p = 0.049; p = 0.147 after Sidak correction for multiple testing). In particular, the frequency of the HLA-DQA1*0102 allele was significantly increased in the AAA group (75/360 alleles, 20.8%) compared to the control group (71/522 alleles, 13.6%) in the Belgian population (empirical p = 0.033; asymptotic p = 0.005; Table 1). No significant associations were found in the Canadian population (Tables 1, 2, 3, 4, 5), but this might be due to lower power to detect such associations because of smaller sample size.

**Table 1:** HLA-DQA1 allele frequencies in AAA cases and controls

	AAA Cases						Controls					
		
	Male		Female		Total		Male		Female		Total	
						
DQA1 alleles	n^a^	%	n^a^	%	n^a^	%	n^a^	%	n^a^	%	n^a^	%
Belgian												
*0101/0104	45	14.0	4	10.5	49	13.6	68	19.1	22	13.3	90	17.2
*0102	67	20.8^b^	8	21.1	75	20.8^c^	44	12.4^b^	27	16.3	71	13.6^c^
*0103	25	7.8	1	2.6	26	7.2	36	10.1	8	4.8	44	8.4
*0201	53	16.5	3	7.9	56	15.6	46	12.9	19	11.4	65	12.5
*03011/0302	37	11.5	5	13.2	42	11.7	54	15.2	30	18.1	84	16.1
*03012	0	0	0	0	0	0	0	0	1	0.6	1	0.2
*0401	8	2.5	3	7.9	11	3.1	11	3.1	8	4.8	19	3.6
*0501	87	27.0	14	36.8	101	28.1	97	27.2	49	29.5	146	28.0
*0601	0	0	0	0	0	0	0	0	2	1.2	2	0.4
Total	322	100.0	38	100.0	360	100.0	356	100.0	166	100.0	522	100.0
Canadian												
*0101/0104	46	14.9	7	7.3	53	13.1	12	18.2	42	17.1	54	17.3
*0102	58	18.8	20	20.8	78	19.3	11	16.7	37	15.0	48	15.4
*0103	11	3.6	7	7.3	18	4.6	4	6.1	15	6.1	19	6.1
*0201	48	15.6	17	17.7	65	15.8	17	25.8	34	13.8	51	16.3
*03011/0302	66	21.4	19	19.8	85	21.0	12	18.2	51	20.7	63	20.2
*03012	0	0	0	0	0	0	0	0	0	0	0	0
*0401	7	2.3	2	2.1	9	2.2	1	1.5	9	3.7	10	3.2
*0501	71	23.1	24	25.0	95	23.5	9	13.6	57	23.2	66	21.2
*0601	1	0.3	0	0	1	0.2	0	0	1	0.4	1	0.3
Total	308	100.0	96	100.0	404	100.0	66	100.0	246	100.0	312	100.0

AAA: abdominal aortic aneurysm.

^a ^Number of alleles.

^b ^Empirical P = 0.019, asymptotic P = 0.003 for comparison of this particular allele versus all other alleles combined between cases and controls among Belgian males.

^c ^Empirical P = 0.033, asymptotic P = 0.005 for comparison of this particular allele versus all other alleles combined between cases and controls among Belgians.

**Table 2:** HLA-DQB1 allele frequencies in AAA cases and controls

	AAA Cases						Controls					
		
	Male		Female		Total		Male		Female		Total	
						
DQB1 alleles^a^	n^b^	%	n^b^	%	n^b^	%	n^b^	%	n^b^	%	n^b^	%
Belgian												
*0201/0202	62	19.4	5	15.2	67	19.0	72	21.4	36	21.8	108	21.5
*0301	59	18.5	9	27.2	68	19.3	68	20.2	46	27.9	114	22.7
*0302	32	10.0	3	9.1	35	10.0	20	5.9	10	6.1	30	6.0
*03031	0	0	0	0	0	0	7	2.1	2	1.2	9	1.8
*03032	10	3.1	0	0	10	2.8	8	2.4	5	3.0	13	2.6
*0304	8	2.5	3	9.1	11	3.1	7	2.1	4	2.4	11	2.2
*0305	3	0.9	0	0	3	0.9	1	0.3	0	0	1	0.2
*0401	10	3.1	3	9.1	13	3.7	10	3.0	8	4.8	18	3.6
*0501	40	12.5	4	12.1	44	12.5	56	16.6	15	9.1	71	14.1
*0502	7	2.2	0	0	7	2.0	5	1.5	4	2.4	9	1.8
*0503	9	2.8	0	0	9	2.6	8	2.4	7	4.2	15	3.0
*0601	0	0	0	0	0	0	1	0.3	0	0	1	0.2
*0602	41	12.9	1	3.0	42	12.0	24	7.1	14	8.5	38	7.6
*0603	23	7.2	3	9.1	26	7.4	33	9.8	6	3.6	39	7.8
*0604	13	4.1	2	6.1	15	4.3	12	3.6	5	3.0	17	3.4
*0605/0609	1	0.3	0	0	1	0.3	3	0.9	2	1.2	5	1.0
*0606	0	0	0	0	0	0	0	0	1	0.6	1	0.2
*0607	1	0.3	0	0	1	0.3	2	0.6	0	0	2	0.4
Total	319	100.0	33	100.0	352	100.0	337	100.0	165	100.0	502	100.0
Canadian												
*0201/0202	77	25.1	26	25.5	103	25.2	19	28.8	63	25.6	82	26.3
*0301	54	17.6	20	19.6	74	18.1	9	13.6	42	17.1	51	16.3
*0302	35	11.4	11	10.8	46	11.2	8	12.1	30	12.2	38	12.2
*03031	5	1.6	0	0	5	1.2	0	0	1	0.4	1	0.3
*03032	16	5.2	5	4.9	21	5.1	4	6.0	6	2.4	10	3.2
*0304	4	1.3	0	0	4	1.0	0	0	3	1.2	3	1.0
*0305	0	0	0	0	0	0	0	0	0	0	0	0
*0401	6	2.0	1	1.0	7	1.7	1	1.5	8	3.3	9	2.9
*0501	38	12.4	8	7.8	46	11.2	8	12.1	33	13.4	41	13.1
*0502	6	2.0	1	1.0	7	1.7	0	0	0	0	0	0
*0503	8	2.6	1	1.0	9	2.2	3	4.5	9	3.7	12	3.8
*0601	2	0.7	1	1.0	3	0.7	0	0	1	0.4	1	0.3
*0602	31	10.1	18	17.6	49	12.0	9	13.6	25	10.2	34	10.9
*0603	12	3.9	6	5.9	18	4.4	4	6.0	14	5.7	18	5.8
*0604	10	3.3	3	2.9	13	3.2	1	1.5	9	3.7	10	3.2
*0605/0609	3	1.0	1	1.0	4	1.0	0	0	1	0.4	1	0.3
*0606	0	0	0	0	0	0	0	0	0	0	0	0
*0607	0	0	0	0	0	0	0	0	1	0.4	1	0.3
Total	307	100.0	102	100.0	409	100.0	66	100.0	246	100.0	312	100.0

AAA: abdominal aortic aneurysm.

^a ^Alleles *0402, *0504 and *0608 were also assayed but were not present in either population.

^b ^Number of alleles.

**Table 3:** HLA-DRB1 allele frequencies in AAA cases and controls

	AAA Cases						Controls					
		
	Male		Female		Total		Male		Female		Total	
						
DRB1 alleles^a^	n^b^	%	n^b^	%	n^b^	%	n^b^	%	n^b^	%	n^b^	%
Belgian												
*0101	29	9.1	3	8.3	32	9.0	39	10.9	14	8.3	53	10.1
*0102	7	2.2	1	2.8	8	2.2	7	2.0	4	2.4	11	2.1
*0103	1	0.3	1	2.8	2	0.6	0	0	0	0	0	0
*0301/0303	5	1.6	1	2.8	6	1.7	7	2.0	9	5.4	16	3.0
*0302	22	6.9	2	5.6	24	6.7	27	7.5	8	4.8	35	6.7
*0401	11	3.4	3	8.3	14	3.9	20	5.6	10	6.0	30	5.7
*0402	1	0.3	0	0	1	0.3	1	0.3	2	1.2	3	0.6
*0403	7	2.2	2	5.6	9	2.5	13	3.6	5	3.0	18	3.4
*0404	9	2.8	0	0	9	2.5	10	2.8	6	3.6	16	3.0
*0405	0	0	0	0	0	0	4	1.1	0	0	4	0.8
*0407	2	0.6	0	0	2	0.6	2	0.6	1	0.6	3	0.6
*0408	0	0	0	0	0	0	1	0.3	0	0	1	0.2
*0410	1	0.3	0	0	1	0.3	0	0	0	0	0	0
*0413	3	0.9	0	0	3	0.8	1	0.3	1	0.6	2	0.4
*0701/0702	54	16.9	3	8.3	57	16.0	51	14.2	18	10.7	69	13.1
*0801	9	2.8	3	8.3	12	3.4	11	3.1	6	3.6	17	3.2
*0802	0	0	0	0	0	0	1	0.3	2	1.2	3	0.6
*0803	1	0.3	0	0	1	0.3	0	0	2	1.2	2	0.4
*0805	0	0	0	0	0	0	0	0	0	0	0	0
*0901	4	1.3	0	0	4	1.1	5	1.4	2	1.2	7	1.3
*1001	3	0.9	0	0	3	0.8	7	2.0	0	0	7	1.3
*1101	41	12.8	8	22.2	49	13.8	38	10.6	25	14.9	63	12.0
*1102	0	0	0	0	0	0	2	0.6	1	0.6	3	0.6
*1103	7	2.2	1	2.8	8	2.2	4	1.1	1	0.6	5	1.0
*1104	1	0.3	0	0	1	0.3	1	0.3	0	0	1	0.2
*1201	3	0.9	1	2.8	4	1.1	6	1.7	0	0	6	1.1
*1301	14	4.4	3	8.3	17	4.8	10	2.8	1	0.6	11	2.1
*1302	18	5.6	1	2.8	19	5.3	34	9.5	13	7.7	47	8.9
*1303	3	0.9	0	0	3	0.8	3	0.8	4	2.4	7	1.3
*1305	0	0	0	0	0	0	0	0	0	0	0	0
*1401	5	1.6	0	0	5	1.4	5	1.4	1	0.6	6	1.1
*1407	3	0.9	0	0	3	0.8	6	1.7	3	1.8	9	1.7
*1501	0	0	0	0	0	0	1	0.3	2	1.2	3	0.6
*1502	1	0.3	0	0	1	0.3	0	0	0	0	0	0
*1601	47	14.7	2	5.6	49	13.8	27	7.5	19	11.3	46	8.7
*1602	0	0	0	0	0	0	2	0.6	0	0	2	0.4
*0301/0303 or *0302	1	0.3	0	0	1	0.3	2	0.6	3	1.8	5	1.0
*0403 or *0407	0	0	0	0	0	0	1	0.3	1	0.6	2	0.4
*0404 or *0408	0	0	0	0	0	0	0	0	1	0.6	1	0.2
*1101 or *1104	0	0	0	0	0	0	0	0	0	0	0	0
*1301 or *1302	7	2.2	1	2.8	8	2.2	5	1.4	2	1.2	7	1.3
*1401 or *1407	0	0	0	0	0	0	2	0.6	1	0.6	3	0.6
CND	0	0	0	0	0	0	2	0.6	0	0	2	0.4
Total	320	100.0	36	100.0	356	100.0	358	100.0	168	100.0	526	100.0
Canadian												
*0101	27	8.8	7	7.0	34	8.3	4	6.1	21	8.5	25	8.0
*0102	1	0.3	1	1.0	2	0.5	2	3.0	4	1.6	6	1.9
*0103	6	1.9	1	1.0	7	1.7	2	3.0	4	1.6	6	1.9
*0301/0303	13	4.2	2	2.0	15	3.7	3	4.5	7	2.8	10	3.2
*0302	26	8.4	12	12	38	9.3	2	3.0	23	9.3	25	8.0
*0401	31	10.0	8	8.0	39	9.6	6	9.1	28	11.3	34	10.8
*0402	2	0.6	0	0	2	0.5	0	0	0	0	0	0
*0403	8	2.6	7	7.0	15	3.7	3	4.5	7	2.8	10	3.2
*0404	17	5.5	4	4.0	21	5.1	3	4.5	11	4.4	14	4.5
*0405	0	0	0	0	0	0	0	0	2	0.8	2	0.6
*0407	1	0.3	2	2.0	3	0.7	0	0	0	0	0	0
*0408	1	0.3	0	0	1	0.2	0	0	0	0	0	0
*0410	0	0	0	0	0	0	0	0	0	0	0	0
*0413	0	0	0	0	0	0	0	0	0	0	0	0
*0701/0702	49	15.9	18	18	67	16.4	18	27.3	36	14.5	54	17.2
*0801	7	2.3	1	1.0	8	2.0	0	0	6	2.4	6	1.9
*0802	0	0	1	1.0	1	0.2	0	0	2	0.8	2	0.6
*0803	0	0	0	0	0	0	0	0	1	0.4	1	0.3
*0805	0	0	0	0	0	0	1	1.5	0	0	1	0.3
*0901	7	2.3	0	0	7	1.7	0	0	2	0.8	2	0.6
*1001	3	1.0	0	0	3	0.7	0	0	3	1.2	3	1.0
*1101	22	7.1	7	7.0	29	7.1	3	4.5	21	8.5	24	7.6
*1102	1	0.3	0	0	1	0.2	0	0	1	0.4	1	0.3
*1103	1	0.3	1	1.0	2	0.5	0	0	0	0	0	0
*1104	0	0	0	0	0	0	0	0	0	0	0	0
*1201	0	0	1	1.0	1	0.2	0	0	3	1.2	3	1.0
*1301	8	2.6	3	3.0	11	2.7	1	1.5	1	0.4	2	0.6
*1302	12	3.9	8	8.0	20	4.9	3	4.5	21	8.5	24	7.6
*1303	3	1.0	0	0	3	0.7	0	0	0	0	0	0
*1305	1	0.3	0	0	1	0.2	0	0	1	0.4	1	0.3
*1401	5	1.6	0	0	5	1.2	2	3.0	2	0.8	4	1.3
*1407	2	0.6	0	0	2	0.5	1	1.5	6	2.4	7	2.2
*1501	0	0	0	0	0	0	0	0	0	0	0	0
*1502	0	0	0	0	0	0	0	0	0	0	0	0
*1601	42	13.6	15	15	57	14.0	9	13.6	29	11.7	38	12.1
*1602	0	0	0	0	0	0	0	0	0	0	0	0
*0301/0303 or *0302	4	1.3	0	0	4	1.0	1	1.5	2	0.8	3	1.0
*0403 or *0407	1	0.3	0	0	1	0.2	0	0	0	0	0	0
*0404 or *0408	0	0	1	1.0	1	0.2	0	0	0	0	0	0
*1101 or *1104	1	0.3	0	0	1	0.2	0	0	0	0	0	0
*1301 or *1302	4	1.3	0	0	4	1.0	1	1.5	1	0.4	2	0.6
*1401 or *1407	1	0.3	0	0	1	0.2	1	1.5	2	0.8	3	1.0
CND	1	0.3	0	0	1	0.2	0	0	1	0.4	1	0.3
Total	308	100.0	100	100.0	408	100.0	66	100.0	248	100.0	314	100.0

AAA: abdominal aortic aneurysm; CND: could not be determined.

^a ^Alleles *0406, *0409, *0411, *0412, *0414, *0415, *0804, *0806, *1105, *1202, *1304, *1306, *1402, *1403, *1404, *1405, *1406, *1408, *1409,*1410 and *1503 were also assayed but were not present in either population.

^b ^Number of alleles.

^c ^Could be classified into two possible alleles, but could not be classified into one particular allele.

**Table 4:** HLA-DRB3-5 allele frequencies in AAA cases and controls

	AAA Cases						Controls					
		
	Male		Female		Total		Male		Female		Total	
						
DRB3-5 alleles	n^a^	%	n^a^	%	n^a^	%	n^a^	%	n^a^	%	n^a^	%
Belgian												
B3*0101	40	12.5	8	22.2	48	13.5	53	14.8	24	14.4	77	14.6
B3*0201	11	3.4	1	2.8	12	3.4	11	3.1	0	0	11	2.1
B3*0202	63	19.7	8	22.2	71	19.9	86	24.0	35	21.0	121	23.0
B3*0301	34	10.6	7	19.4	41	11.5	45	12.5	22	13.2	67	12.7
B4*0101	94	29.4	7	19.4	101	28.4	103	28.7	52	31.1	155	29.5
B5*0101	50	15.6	2	5.6	52	14.6	31	8.6	16	9.6	47	8.9
B5*0102	4	1.3	0	0	4	1.1	2	0.6	3	1.8	5	1.0
B5*0201	7	2.2	0	0	7	2.0	6	1.7	1	0.6	7	1.3
B5*0203	0	0	0	0	0	0.0	0	0	0	0	0	0
B3*0201 or *0202 ^b^	13	4.1	1	2.8	14	3.9	20	5.6	10	6.0	30	5.7
CND	4	1.3	2	5.6	6	1.7	2	0.6	4	2.4	6	1.1
Total	320	100.0	36	100.0	356	100.0	359	100.0	167	100.0	526	100.0
Canadian												
B3*0101	53	17.2	17	17	70	17.2	9	13.6	38	15.3	47	15.0
B3*0201	9	2.9	5	5.0	14	3.4	0	0	6	2.4	6	1.9
B3*0202	44	14.3	15	15	59	14.5	8	12.1	46	18.5	54	17.2
B3*0301	38	12.3	13	13	51	12.5	11	16.7	32	12.9	43	13.7
B4*0101	108	35.1	32	32	140	34.3	26	39.4	80	32.3	106	33.8
B5*0101	45	14.6	14	14	59	14.5	12	18.2	38	15.3	50	15.9
B5*0102	1	0.3	1	1.0	2	0.5	0	0	3	1.2	3	1.0
B5*0201	2	0.6	1	1.0	3	0.7	0	0	1	0.4	1	0.3
B5*0203	1	0.3	0	0	1	0.2	0	0	0	0	0	0
B3*0201 or *0202 ^b^	3	1.0	0	0	3	0.7	0	0	0	0	0	0
CND	4	1.3	2	2.0	6	1.5	0	0	4	1.6	4	1.3
Total	308	100.0	100	100.0	408	100.0	66	100.0	248	100.0	314	100.0

AAA: abdominal aortic aneurysm; CND: could not be determined.

^a ^Number of alleles.

^b ^Could be classified into HLA-DRB*0201 or *0202, but could not be classified into one particular allele.

**Table 5:** Empirical and asymptotic p-values for comparisons between AAA cases and controls within each population and stratified by sex

Locus	Belgian						Canadian					
		
	Male^a^		Female^a^		Total		Male^a^		Female^a^		Total	
						
	EP^b^	AP^c^	EP^b^	AP^c^	EP^b^	AP^c^	EP^b^	AP^c^	EP^b^	AP^c^	EP^b^	AP^c^
DQA1	0.027	0.071	0.899	0.900	0.039	0.049	0.392	0.487	0.340	0.443	0.589	0.682
DQB1	0.083	0.130	0.495	0.710	0.129	0.144	0.917	0.972	0.405	0.553	0.371	0.510
DRB1	0.252	0.375	0.218	0.653	0.114	0.200	0.465	0.890	0.408	0.864	0.246	0.545
DRB3-5	0.145	0.217	0.239	0.308	0.217	0.308	0.745	0.778	0.926	0.952	0.766	0.739

AAA: abdominal aortic aneurysm; EP: empirical p-value; AP: asymptotic p-value.

^a ^Number of Belgian AAA cases: Male = 161, Female = 19; Canadian AAA cases: Male = 155, Female = 52; Belgian controls: Male = 178, Female = 91; Canadian controls: Male = 39, Female = 118.

^b ^Global empirical p-values were calculated by permutation test using the CLUMP [28] program.

^c ^Asymptotic p-values were calculated by χ^2 ^test.

The results on analyses in which the AAA cases and controls were stratified by sex are shown in Table 5. The HLA-DQA1 allele frequencies in AAA cases and controls in the Belgians stratified by sex are shown in Table 1. The association between AAA and HLA-DQA1 locus remained nominally significant only in the Belgian male population (empirical p = 0.027; asymptotic p = 0.071; p = 0.104 after Sidak correction for multiple testing). There was a significant increase in the frequency of the HLA-DQA1*0102 allele among AAA cases (67/322 alleles, 20.8%) compared to controls (44/356 alleles, 12.4%) in the Belgian male population (empirical p = 0.019; asymptotic p = 0.003). The odds ratio was 1.86 and 95% confidence interval was 1.56 to 2.22 for the HLA-DQA1*0102 allele versus all the other alleles in the Belgian males. Since the number of female cases was small, no conclusions can be drawn about the association in females. In the Belgian population, the association between AAA and DQA1 was greater among those with a family history of AAA (empirical p = 0.069; asymptotic p = 0.067) compared to those without a family history of AAA (empirical p = 0.157; asymptotic p = 0.164). Since the proportion of males was similar in the group with (88%) and without (90%) family history, the result was not due to difference in the number of males in each group. No additional significant associations were found between AAA cases and controls when AAA cases were stratified by family history of AAA (Table 6).

**Table 6:** Analysis of HLA loci in AAA cases and controls when stratified by family history of AAA

Locus	Belgian				Canadian			
		
	Family history		No family history		Family history		No family history	
				
	EP^a^	AP^b^	EP^a^	AP^b^	EP^a^	AP^b^	EP^a^	AP^b^
DQA1	0.069	0.067	0.157	0.164	0.198	0.284	0.764	0.832
DQB1	0.419	0.415	0.305	0.317	0.384	0.532	0.432	0.571
DRB1	0.597	0.811	0.099	0.171	0.232	0.545	0.242	0.659
DRB3-5	0.356	0.454	0.314	0.414	0.831	0.796	0.490	0.581

AAA: abdominal aortic aneurysm; EP: empirical p-value; AP: asymptotic p-value.

^a ^Global empirical p-values were calculated by permutation test using the CLUMP [28] program.

^b ^Asymptotic p-values were calculated by χ^2 ^test.

The results of haplotype analyses for the HLA-DQA1, -DQB1, -DRB1 and -DRB3-5 loci between AAA cases and controls are shown in Tables 7 and 8. Only the most significant haplotypes and their p-values when analyzing two-locus haplotypes are listed. Although most of the frequencies of the most significant haplotypes were relatively rare (< 5%), haplotype DQA1*0102/DRB1*1601 had a relatively high frequency (11.6%; Table 7). Using the global score statistics, the nominal p-value was 0.049 and with haplotype-specific score statistics, the nominal p-value was 0.002 for this haplotype, which is not as strong statistical evidence as the result for the DQA1 locus alone. In addition, the haplotype results were not significant among the Belgian males (Table 8), as was observed for DQA1 alone.

**Table 7:** Haplotype analyses when comparing two HLA loci

Haplotype combination	Global Score Statistics	DF	p value	Most significant haplotype		Haplotype-specific score statistics^a^	p value^a^
						
				Haplotype	Population Frequency (%)		
DQA1+DQB1	62.7	58	0.311	0201~0302	1.2	3.01	0.003
DQA1+DRB1	91.8	71	0.049	0102~1601	11.6	3.04	0.002
DQA1+DRB3-5	50.6	51	0.491	0101~B3-0202	4.8	-2.39	0.017
DQB1+DRB1	125.7	119	0.319	0604~1301	1.1	2.36	0.018
DQB1+DRB3-5	66.2	77	0.806	0301~B3-0201	1.9	2.02	0.044
DRB1+DRB3-5	115.1	114	0.453	1601~B5-0101	10.3	2.35	0.019

DF: degrees of freedom; AAA: abdominal aortic aneurysm.

^a ^Haplo.stats program [29] was used for the analysis. Rare haplotypes with frequencies of ≤ 0.01 were pooled into a single baseline group. Results on haplotypes with only two loci are reported.

**Table 8:** Haplotype analyses for two HLA loci combined and stratified by population and sex

Population	Sex	Haplotype combination	Global Score Statistics	DF	p value	Most significant haplotype		Haplotype-specific score statistics^a^	p value^a^
								
						Haplotype	Population frequency (%)		
Belgian									
	Male	DQA1+DQB1	54.0	42	0.102	0102~0602	9.4	2.97	0.003
		DQA1+DRB1	57.8	53	0.303	0102~1601	10.5	3.50	0.001
		DQA1+DRB3-5	44.8	39	0.240	0102~B5-0101	9.4	2.95	0.003
		DQB1+DRB1	81.1	82	0.508	0602~1601	8.9	2.88	0.004
		DQB1+DRB3-5	53.5	60	0.710	0602~B5-0101	9.2	2.88	0.004
		DRB1+DRB3-5	84.5	81	0.374	1601~B5-0101	9.3	3.24	0.001
									
	Female	DQA1+DQB1	29.7	28	0.380	0102~0603	1.3	3.48	0.001
		DQA1+DRB1	41.6	34	0.173	0102~1301	2.1	3.52	0.001
		DQA1+DRB3-5	22.9	26	0.638	0501~B3-0201	0.6	2.12	0.033
		DQB1+DRB1	40.7	49	0.794	0604~1301	2.2	2.81	0.005
		DQB1+DRB3-5	38.0	37	0.422	0603~B5-0101	0.6	2.74	0.006
		DRB1+DRB3-5	49.4	48	0.418	1301~B3-0301	2.2	2.92	0.003
									
Canadian									
	Male	DQA1+DQB1	27.6	32	0.691	0101~0201	0.3	-2.16	0.031
		DQA1+DRB1	36.8	38	0.527	0101~0102	0.8	-2.34	0.020
		DQA1+DRB3-5	27.8	33	0.724	0401~B5-0101	0.3	-2.00	0.046
		DQB1+DRB1	55.7	56	0.486	0501~0102	0.8	-2.22	0.027
		DQB1+DRB3-5	36.3	49	0.910	03032~B3-0301	1.1	-3.32	0.001
		DRB1+DRB3-5	55.6	63	0.735	0701~B4-0101	11.4	-2.19	0.028
									
	Female	DQA1+DQB1	29.1	31	0.563	0201~0302	1.2	2.01	0.045
		DQA1+DRB1	41.4	38	0.326	0103~1301	0.4	2.25	0.024
		DQA1+DRB3-5	34.1	33	0.414	0102~B3-0101	0.6	2.37	0.018
		DQB1+DRB1	69.6	62	0.237	0302~0407	0.5	2.71	0.007
		DQB1+DRB3-5	49.0	43	0.245	0602~B4-0101	0.9	2.93	0.003
		DRB1+DRB3-5	61.1	60	0.437	0301~B3-0301	0.2	2.37	0.017

DF: degrees of freedom; AAA: abdominal aortic aneurysm.

^a ^Haplo.stats program [29] was used for the analyses. Rare haplotype with frequencies ≤ 0.01 were pooled into a single baseline group. Results on haplotypes with only two loci are reported.

## Discussion

AAA is a relatively common disease in that an estimated 1–6% of the population in industrialized countries harbor aneurysms [32]. Rupture of AAAs causes 1–2% of all deaths in males over 65 years of age in Western countries [33,34]. It has been suggested that AAAs are a complex disease [35,36]. Two segregation studies favored a genetic model in explaining the familial aggregation of AAA and suggested the presence of a major gene effect [35,36]. Recently, we reported on a collection of 233 families with at least two individuals affected with AAA [37], and identified two genetic susceptibility loci for AAA on chromosomes 19q13 and 4q31 [38]. We also recently reported a genetic association study for polymorphisms in biologically relevant candidate genes for AAA, and found evidence for an association between tissue inhibitor of metalloproteinases 1 (TIMP1) polymorphisms and AAA [31].

It is well known that there are large differences in the frequencies of the HLA polymorphisms in different populations. We, therefore, carried out the analyses with Belgian and Canadian populations separately, to avoid potential false positive results because of population stratification. There have, however, been several examples where substructure within Caucasian samples has been explored and identified [39,40], thus the possibility of cryptic sub-structure within each population remains a concern. Although ancestry informative marker (AIM) panels exist, these panels have been created with the intent of distinguishing the major racial/ethnic groups, and may not be sensitive for detecting population substructure within a single racial/ethnic group. Ideally, it will be possible in the future to utilize AIM panels that are specific to addressing the question of cryptic relatedness within the Caucasian population.

We observed a potential association between the HLA-DQA1 locus and AAA among the Belgian males. In particular, there was a statistically significant difference in the frequency of the HLA-DQA1*0102 allele between AAA cases and controls in the Belgian males. This finding suggests that the HLA-DQA1 locus harbors a genetic risk factor for AAAs. Since the number of the Belgian female cases was small (n = 20), further work with a larger sample will be needed to investigate the role of HLA in female AAA cases. No significant associations were observed in the Canadian population, although the frequency of the DQA1*0102 allele was also higher in the Canadian cases than the controls.

Several association studies between HLA polymorphisms and AAA have been performed previously. Most of the previous studies were small and different methods to type the HLA alleles were used making it difficult to compare these studies to each other (Table 9) [11-19]. The most common finding was an association between AAA and HLA-DRB1*02 alleles, which are divided into -DRB1*15 and *16 alleles. Tilson et al [12] demonstrated this association in 26 African-Americans. Rasmussen et al [13,16,17] reported a similar result in 37 North Americans and Hirose et al [14] reported this association in 46 Japanese. Sugimoto et al [18] (n = 49) and Moňux et al [19] (n = 72), however, failed to find such an association in their studies. Our study showed a marginally significant association between haplotype DQA1*0102/DRB1*1601 and AAA. The HLA-DRB1*1601 allele, however, was not significantly associated with AAA by permutation test (empirical p = 0.253; asymptotic p = 0.02). There was no significant association between HLA-DRB1*04 alleles and AAA in the current study, which differs from the results by Rasmussen et al [13] and Moňux et al [19] when using 37 and 72 AAA cases, respectively. No prior association studies between AAA and HLA-DQA1 locus were performed (Table 9).

**Table 9:** Previous HLA studies on AAA

Study	Country	n (Inflammatory AAA)^a^	HLA locus studied	Significant allele^b^	p
Norrgård *et al *1984 [11]	Sweden	48	HLA-A, -B^c^	None	N/A
Tilson *et al *1996 [12]	US	26	HLA-DRB1	DRB1*02	0.04
				DRB1*12	0.02
Rasmussen *et al *1997 [13]	US	37	HLA-DQB1, DRB1	DRB1*15	<0.05
		(37)		DRB1*0404	<0.05
Hirose *et al *1998 [14]	Japan	46	HLA-DR^c^	DR2(15)	<0.005
Hirose & Tilson 1999 [15]	Japan	36	HLA-DQ^c^	DQ3	0.014
Rasmussen *et al *2001 [16]	US	142	HLA-DRB1	DRB1*02	0.03
		(40)			0.01^d^
Rasmussen *et al *2002 [17]	US	142	HLA-DRB1	DRB1*02	<0.01
				DRB1*08	0.04
				DRB1*14	<0.01
Sugimoto *et al *2003 [18]	Japan	49	HLA-A, -B, -DR	A2	<0.05
				B61	<0.005
Moňux *et al *2003 [19]	Spain	72	HLA-DRB1	DRB1*0401	0.02

*AAA*: abdominal aortic aneurysm.

^a ^Inflammatory AAA was categorized and analyzed separately.

^b ^Allele which had a significantly different frequency between AAA cases and controls.

^c ^Serological typing was carried out.

^d ^P-value for inflammatory AAA.

The HLA-DQA1*0102 allele, which showed a potential association with AAA in our study, has been found to be associated previously with other diseases. The HLA-DQA1*0102 allele is known to be a protective allele against type 1 diabetes mellitus (DM) and systemic lupus erythematosus, which are classical autoimmune diseases [41,42]. Interestingly, type 2 DM, which is a well-known risk factor for many cardiovascular diseases, is protective against AAA in the U.S. population [23].

Although there is growing evidence of the association between the HLA system and autoimmune diseases, including AAA, the precise disease-causing mechanism is yet to be defined. It is also possible that an association between HLA and disease is only a marker for an undiscovered polymorphism in a linked gene. There are at least 120 additional genes in the MHC region. Most of these additional genes in the HLA class II region are involved in immunological functions that relate to the HLA class I and II genes [7,43]. Because of the high density of potentially important genes in the MHC region, linkage disequilibrium around this region makes it difficult to identify the exact susceptibility gene for a disease.

Limitations of the study were: 1) the statistical power was relatively low (for significance level of 0.05 the power was 0.49 in the Belgian males; 0.14 in the Belgian females; 0.16 in the Canadian males and 0.22 in the Canadian females using the observed allele frequencies), and after correction for multiple testing, none of the results remained significant. Nevertheless, the findings were consistent in the sense that the HLA-DQA1*0102 allele frequency was always higher in the case groups than in the control groups (20.8% vs. 12.4% in the Belgian males; 21% vs. 16% in the Belgian females; 18.8% vs. 16.7 in the Canadian males; and 20.8% vs. 15% in the Canadian females); 2) although the overall sample size was not small, the number of alleles was large leading to relatively small counts for each allele; 3) we did not test the interaction with other loci and environmental factors, such as age, or smoking; and 4) our sampling scheme including spousal controls could lead to false positive results because of the potential effect of the HLA loci on mate selection. The HLA region has been suggested to influence mate selection based on odor differences coded by the MHC genes, with a preference for dissimilar partners, in both mice and humans [44], although these findings are not always consistent. The presence of negative assortative mating could lead to false positive findings when comparing AAA cases to control spouses.

## Conclusion

We found evidence for genetic association between the HLA-DQA1 locus and AAA suggesting that this genomic region harbors a genetic risk factor for AAAs in the Belgian male population. The same DQA1*0102 allele was present at higher frequency among the Belgian female, Canadian male and Canadian female cases supporting the hypothesis that autoimmunity contributes to the pathogenesis of AAAs. In future studies, the findings need to be assessed in another, larger sample.

## Competing interests

The author(s) declare that they have no competing interests.

## Authors' contributions

TO carried out the molecular assays, scored results and prepared the manuscript. LG designed the study and scored results. KG designed and carried out statistical analyses as well as prepared the manuscript. MS carried out the molecular assays. GT designed the study and analyzed data. WL designed the study and analyzed data. AP carried out statistical analyses. QL carried out statistical analyses. HS designed the study and carried out some of the molecular assays. NS recruited Belgian patients and controls into the study. RL recruited Belgian patients and controls into the study. GM recruited Canadian patients and controls into the study. CA recruited Canadian patients and controls into the study. TS provided clinical expertise. HK designed the study, analyzed data and prepared the manuscript. All authors gave final approval to the manuscript.

## Pre-publication history

The pre-publication history for this paper can be accessed here:


